# Protective effect of creatine on amikacin-induced ototoxicity

**DOI:** 10.1016/j.bjorl.2020.09.002

**Published:** 2020-10-04

**Authors:** Emre Apaydın, Elif Dağlı, Sevinç Bayrak, Ekrem Said Kankılıç, Hasan Şahin, Aydın Acar

**Affiliations:** a Kecioren Training and Research Hospital, Department of Otolaryngology, Ankara, Turkey; b Guven Private Hospital, Department of Audiology, Ankara, Turkey

**Keywords:** Amikacin, Ototoxicity, Antioxidants, Creatine monohydrate

## Abstract

**Introduction:**

Aminoglycosides are widely known for their ototoxic side effects. Nevertheless, they are potent antibiotics used in the treatment of life-threatening conditions because of the current concern for antibiotic resistance. We hypothesized that creatine supplements which are believed to improve mitochondrial antioxidant defense system and maintain optimal energy homeostasis may improve the ototoxic side effects.

**Objective:**

This study aimed to investigate the protective effects of creatine monohydrate against ototoxicity induced by amikacin in rats in an experimental animal model, using distortion product otoacoustic emissions and auditory brainstem response.

**Methods:**

Twenty healthy rats were assigned to four groups (5 rats in each): the control group, the creatine monohydrate group, the amikacin group and the amikacin + creatine monohydrate group. The creatine monohydrate group received creatine at a dose of 2 g/kg once daily via gastric gavage for 21 days. The amikacin group received amikacin at a dose of 600 mg/kg by intramuscular injections once daily for 21 days. The amikacin + creatine monohydrate group received intramuscular injections of amikacin (600 mg/kg) once daily for 21 days and creatine monohydrate (2 g/kg) once daily via gastric gavage for 21 days. The control group received nothing. The distortion product otoacoustic emissions and auditory brainstem response measurements were performed on all rats on days 0, 7, 21.

**Results:**

Regarding auditory brainstem response values, a significant increase in the auditory threshold was observed in the amikacin group on day 21 (*p* <  0.001). The amikacin+creatine monohydrate group showed significantly lower levels of auditory brainstem response auditory thresholds on day 21 in comparison to the amikacin group (*p* <  0.001). Additionally, the control group and the amikacin+creatine monohydrate group did not differ significantly with respect to auditory brainstem response thresholds on treatment day 21 (*p* >  0.05). When we compare distortion product otoacoustic emissions values, there was no significant difference between the amikacin and amikacin+creatine monohydrate groups on day 7 (*p* >  0.05), However significantly greater distortion product otoacoustic emissions values were observed in the amikacin+creatine monohydrate group on day 21 compared to the amikacin group (*p* <  0.001).

**Conclusion:**

Our findings demonstrate that creatine treatment protects against amikacin ototoxicity when given at a sufficient dose and for an adequate time period.

## Introduction

The toxic effect of some therapeutic drugs on the auditory and vestibular functions is defined as drug-induced ototoxicity.^1^ Aminoglycosides (AG) are broad-spectrum antibiotics that have long been used widely for the treatment of gram-negative bacterial infections, primarily tuberculosis.^2^, ^3^

Amikacin, the first semi-synthetic derivative of AG, is produced from the natural drug kanamycin by acetylation.^4^

Among AGs, amikacin, kanamycin and neomycin mainly cause cochlear damage, whereas streptomycin and gentamicin are mostly associated with vestibular damage.^5^ Cochlear toxicity induced by amikacin occurs through apoptosis as a result of generation of reactive oxygen species or free oxygen radicals by amikacin. Consequently, ototoxicity and deafness develop.^6^

Creatine (Cr) is a natural substance and an important source for cellular energy which can be synthesized endogenously in humans and rodents^7^ but it may also be taken exogenously in the diet. Recent studies have demonstrated antioxidant activity of creatine.^8^

The role of Cr in the energy metabolism and its neuroprotective action in neurodegenerative disorders due to its antioxidant properties have been reported in recent literature.^9^, ^10^, ^11^ In addition, it was suggested that Cr may be effective in the treatment of hearing loss in Huntington’s disease, which is a neurodegenerative disease,^12^ and favorable effects of Cr treatment on noise-induced hearing loss have been demonstrated in an animal model.^13^

In the present study, we aimed to investigate protective effects of Cr against ototoxicity caused by free oxygen radicals generated by amikacin using the Distortion Product Otoacoustic Emissions (DPOAEs) and the Auditory Brainstem Response (ABR).

## Methods

### Animals

For this study, 20 female adult Wistar albino rats (body weight, 200–240 g each) were kept in plastic cages under standardized housing conditions of a 12/12 hours of dark-light cycle at a temperature of 21 °C, with free access to food and water. The animals were used in accordance with the Guide for the Care and Use of Laboratory Animals.^14^ All study rats underwent otomicroscopic ear examination (Zeiss, OPMI 9, Göttingen, Germany) prior to the audiological tests and rats with an external or middle ear pathology were excluded. Distortion Product Otoacoustic Emissions (DPOAEs) and Auditory Brainstem Response (ABR) recordings were performed in a quiet room. The experimental design was approved by the Ethics Committee for Research on Animals of Ankara Research and Training Hospital (approval number 0046). The study was performed between 2018 August and 2018 September at the animal experimentation laboratory of Ankara Research and Training Hospital.

### Anesthesia

Ketamine (100 mg/mL; 0.1 mL/100 g) (Ketalar®50 mg/mL 10 mL, Pfizer, Istanbul, Turkey) and xylazine (20 mg/mL; 0.025 mL/100 g) (Kepro Xylazine®20 Inj. 20 mg/mL 25 mL, BioPharm, Istanbul, Turkey) were used for anesthesia. Anesthetic agents were administered via intraperitoneal injections before the recordings.

### Study design

This was an experimental blinded randomized study such that only the caregivers knew which test subject belong to what group and the researchers were blinded to treatment assignment while performing the audiological examination. Rats were assigned to 4 groups, each having 5 rats: the control group, the Creatine Monohydrate (CrMn) group, the amikacin group and the amikacin + CrMn group. The CrMn group received creatine monohydrate (Sigma Chemical Co, St Louis, MO) at a dose of 2 g/kg once daily via gastric gavage for 21 days. The amikacin group received amikacin (Amikozit® 500 mg, Sanofi Sağlık Ürünleri, İstanbul, Turkey) at a dose of 600 mg/kg by intramuscular injections once daily for 21 days. The amikacin + CrMn group received intramuscular injections of amikacin (600 mg/kg) once daily for 21 days and CrMn (2 g/kg) once daily via gastric gavage for 21 days. The control group did not receive any treatment. The DPOAE and ABR measurements were performed on all rats on days 0, 7, 21. Results obtained before and after treatment were compared for each group and between groups.

### Audiological evaluation

#### Distortion product otoacoustic emission (DPOAE)

An Eclipse 25 ABR System (Interacoustics, Middelfart, Denmark) device was used for DPOAE measurements. The smallest elastic tympanometry probe available was used for the rats. Otoacoustic emissions were measured using stimuli of constant intensity and frequency changes. The frequencies (f1 and f2) were adjusted to the f2:f1 ratio of 1.22 and stimulus intensity levels of L1 = 65 dB, L2 = 55 dB. DP-gram measurements were performed at 1000, 2000, 4000, 6000 Hz frequencies. A signal/noise ratio above (S/N level) 7 dB was the threshold to stop recording. The measurements were recorded from both ears which took a minute to perform.

#### Auditory Brainstem Response (ABR)

An Eclipse 25 ABR System (Interacoustics, Middelfart, Denmark) device was used for ABR measurements. Subdermal needle electrodes (Technomed Europe, Limburg, Netherlands) and ER 3A insert earphones were used to provide click stimuli in alternating polarities. The filter was set at 100, 3000 Hz, repetition rate at 49,1 second, and the time window at 15 milliseconds. The stimuli were presented at 70 dB normal hearing level intensity, and the intensity level was reduced in 10 dB steps until near-threshold values. Then, the intensity level was reduced in 5 dB steps until the threshold value was reached. At least 2 tracks were generated for each measurement to test behavior reproducibility, and the threshold was cross-checked. The electrode placement was as follows: the ground electrode was placed on the back, the positive electrode was on the vertex and one of the negative electrodes was on the left mastoid apex and the other on the right mastoid apex.

### Statistical analyses

An a priori sample size calculation showed that a sample size of 5 rats in each group would be needed for the study to detect a difference between the study groups with a power of 90% and a significance level of 5%. Statistical analyses were performed using the SPSS version 16.0 (SPSS Inc, Chicago, IL, USA). All quantitative variables were estimated using measures of central location (i.e., mean and median) and measures of dispersion (i.e., standard deviation). Data normality was checked using the Shapiro-Wilk test for normality. Parametric tests were employed because of the normal distribution of the values. One-way Analysis of Variance (ANOVA) was used for inter-group comparisons of the DPOAE and ABR values and the statistical significance was set at *p* < 0.001. Tukey’s Honest Significant Difference (HSD) post hoc test was used to determine whether there were differences among the groups; the statistical significance was set at *p* < 0.001 for post hoc tests. Pretreatment-posttreatment (Day 0 vs. Day 21) differences in the DPOAE amplitudes and ABR values in the 4 groups were analyzed using paired samples *t*-tests, with significance set at *p* <  0.001.

## Results

**Figure 1:**
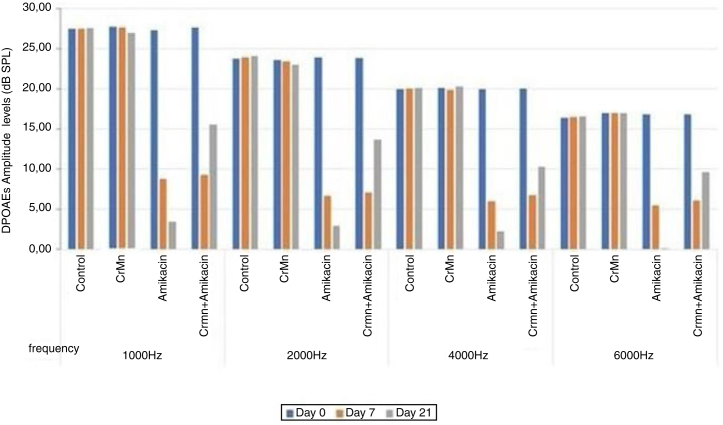
DPOAEs amplitude levels of groups in different frequencies. DPOAE, Amplitude levels of groups at different frequencies.

**Figure 2:**
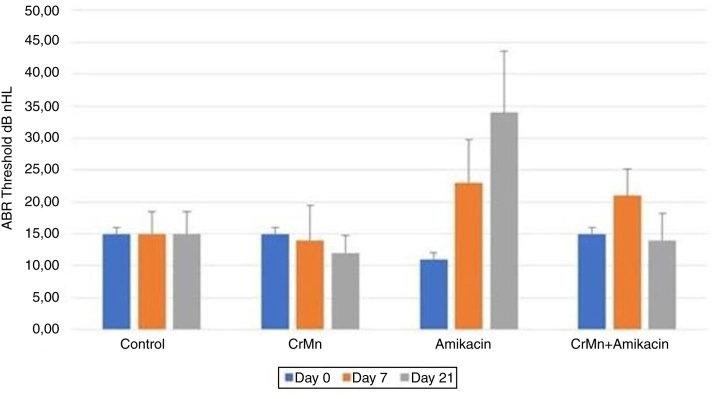
ABR Thresholds of groups.

The rats tolerated anesthesia and amikacin and creatine monohydrate administrations, and no difference was observed in the consumption of water or food among the groups. No statistically significant differences in the DPOAE amplitudes were detected in the intragroup and intergroup analyses at the start of the study (*p* >  0.05). Also, the group averages and the right and left ear values were statistically similar (*p* >  0.05) (Fig. 1). There were no significant differences in DPOAE levels between control and CrMn groups during the treatment (days 7 and 21) (*p* >  0.05) (Table 1). However, the DPOAE amplitude levels were significantly reduced in the amikacin and amikacin + CrMn groups on days 7 and 21 (*p* <  0.001) (Fig. 1). On the other hand, there was no statistically significant difference in DPOAE levels between the amikacin group and the amikacin + CrMn group on day 7, indicating that creatine administration for 7 days did not confer any protection against amikacin-induced ototoxicity (*p* >  0.05). However, longer creatine administration for 21 days provided a significant protective effect against amikacin-induced ototoxicity as shown by the test results of the amikacin+CrMn group (*p* <  0.001) (Table 1, Fig. 1). There were no significant differences between the initial ABR thresholds in the intragroup and intergroup analysis (*p* >  0.05) (Fig. 2). No significant difference was observed in ABR thresholds of any group according to the intergroup analysis on day 7 (*p* >  0.05) (Table 2). There was a significant increase in the day 21 thresholds in the amikacin only group (*p* <  0.001) (Fig. 2). The increase in the ABR threshold significantly improved in the amikacin + CrMn group compared to the amikacin only group on day 21 (*p* <  0.001) (Table 2). Moreover, the ABR thresholds of the control group and amikacin + CrMn group were not significantly different on day 21 (*p* >  0.05).

**Table 1:** Intergroup analysis of DPOAE amplitude levels during treatment.

Groups	Day 7	Day 21
Control/CrMn	0.815	0.618
Control/Amikacin	0.000^a^	0.000^a^
Control/Amikacin + CrMn	0.000^a^	0.000^a^
CrMn/Amikacin	0.000^a^	0.000^a^
CrMn/CrMn + Amikacin	0.000^a^	0.000^a^
Amikacin/CrMn + Amikacin	0.995	0.000^a^

*Statistical differences in DPOAE amplitude levels at 6000 Hz among groups as detected by Tukey’s honest significant difference (HSD) post hoc test.

a *p* <  0.001 indicates a significant difference in the DPOAE values among the groups.

**Table 2:** Intergroup analysis of ABR thresholds during treatment.

Groups	Day 7	Day 21
Control/CrMn	0.989	0.839
Control/Amikacin	0.104	0.00^a^
Control/Amikacin + CrMn	0.287	0.992
CrMn/Amikacin	0.059	0.000^a^
CrMn/CrMn + Amikacin	0.177	0.944
Amikacin/CrMn + Amikacin	0.925	0.000^a^

*Statistical differences in ABR thresholds among groups as detected by Tukey’s Honest Significant Difference (HSD) post hoc test.

a *p* <  0.001 indicates a significant difference in the ABR values among the groups.

## Discussion

In the present study, we investigated the potential protective effects of Cr against ototoxicity following exposure to amikacin in an experimental model in rats. Our findings confirmed ototoxicity induced by amikacin using DPOAEs and ABR and demonstrated the protective effect of Cr against amikacin ototoxicity. To our best knowledge, this is the first study in the literature to report on the protective effect of Cr against ototoxicity. Aminoglycosides (AG) are potent antibiotics used in the treatment of infections caused by gram-negative bacteria and tuberculosis; however, the therapeutic use of AG is limited by their side effects including ototoxicity and nephrotoxicity.^4^ Despite being associated with side effects, AGs are still indispensable in the treatment of life- threatening conditions because of the current concern for antibiotic resistance.

Amikacin is used for the treatment of infections caused by microorganisms resistant to other AG antibiotics.^15^ Amikacin is also used for treatment of severe infections due to its synergistic action with certain antibiotics.^16^ We chose amikacin for the present study based on its widespread use.

Amikacin causes apoptosis of outer hair cells in the cochlea through free oxygen radicals.^3^, ^6^ This kind of damage induced by amikacin initially occurs in the base of cochlea and progresses to the apex. Hearing loss as well as reduced Speech Discrimination Scores (SDS) occur as a result of this apoptotic process involving the entire auditory pathway.^17^ Previous studies have shown that amikacin may induce ototoxicity at a dose of 600 mg/kg/day.^17^

In Avcı et al.’s study, rats were given a 600 mg/kg/day dose of amikacin which resulted in a marked ototoxicity during 21 days of follow-up.^18^ In the current study, we used amikacin at the same dose. The statistically significant changes in DPOAE and ABR results observed in the amikacin group at the end of 21 days (*p* <  0.001) showed that this dose is sufficient to induce ototoxicity in rats (Figure 1, Figure 2).

In the current study, we investigated the protective effect of Cr with known antioxidant properties^8^ against ototoxicity induced by oxidant action of amikacin. Creatine is a nitrogen organic acid which is known to increase muscle mass and performance, prevent muscle atrophy caused by diseases and help provide energy to cells via a reversible reaction catalyzed by the enzyme creatine kinase.^12^

In addition to its role in increasing the energy stores, Cr also exhibits a potent antioxidant action by reducing the mitochondrial production of Reactive Oxygen Species (ROS), raising and restoring mitochondrial membrane potential.^19^ Creatine supplements are believed to improve mitochondrial antioxidant defense system and maintain optimal energy homeostasis.^12^

In an animal model of noise-induced hearing loss, Cr treatment was shown to markedly reduce auditory threshold shifts.^13^ The use of creatine for hearing loss induced by this kind of oxidative stress has been considered to be beneficial and necessary due to its role in maintaining ATP levels and its capacity to scavenge free oxygen radicals.^12^

It was shown in rats that Cr supplementation given in the form of CrMn at a dose of 2 g/kg for a period of 2 − 4 weeks is needed to observe any therapeutic efficacy.^20^ Therefore, we used this treatment strategy in the present study.

While hearing was evaluated only by OAE in some studies using experimental animal models^18^, ^21^, ^22^ others used ABR alone.^23^, ^24^ In our study we used both ABR and DPOAE measurements in order to improve the reliability of our results.^25^, ^26^, ^27^, ^28^

In the present study, significantly lower DPOAE values were observed on days 7 and 21 in the groups given amikacin and amikacin + CrMn compared to baseline (*p* <  0.001) (Fig. l). On day 21, amikacin+CrMn group showed significantly greater DPOAE values compared to day 7 (Fig. 1). While there was no significant difference in the DPOAE values between amikacin and amikacin + CrMn groups on day 7 (*p* >  0.05), significantly greater DPOAE values were observed in the amikacin + CrMn group on day 21 compared to the amikacin group (*p* <  0.001) (Fig. 1).

Damage to the ear caused by amikacin may progress to the eighth cranial nerve. Regarding ABR values, a significant increase in the auditory thresholds was only observed in the amikacin group on day 21 (*p* <  0.001) (Fig. 2). The amikacin + CrMn group showed significantly lower levels of auditory thresholds on day 21 in comparison to the amikacin group (*p* <  0.001) (Table 2). Additionally, the control group and the amikacin + CrMn group did not differ significantly with respect to ABR thresholds on treatment day 21 (*p* >  0.05) (Table 2). In light of these findings, it may be concluded that Cr supplementation does not confer a protective effect against ototoxicity when given for 7 days and its protective effect occurs after 21 days of treatment. Our findings are consistent with a previous study which demonstrated the efficacy of Cr in rats following a treatment period of 2 − 4 weeks.^20^ Considering ABR and DPOAE values, no statistically significant difference was observed between control group and CrMn only group on days 0, 7 and 21 (*p* >  0.05) (Table 1, Table 2), suggesting that Cr is not ototoxic.

In a study using an experimental animal model, Aksoy et al. reported that ototoxic effects of amikacin may be limited by concomitant use of thymoquinone. In addition to ABR and DPOAE measurements, the same authors assessed oxidative stress markers of Total Antioxidant Status (TAS) and Total Oxidant Status (TOS)^27^ and showed that amikacin increases oxidative stress using these measurements. There are studies in the literature that reported the protective effects of resveratrol, trimetazidine, thymoquinone and pentoxifylline against amikacin-induced ototoxicity, all of which have demonstrated antioxidant efficacy.^18^, ^21^, ^27^, ^29^

Although our study showed the protective effect of creatine on amikacin ototoxicity based on ABR and DPOAE results, it is not a sufficient indicator of functional and histological damage. Therefore, the cochlea should be studied histologically in order to definitively demonstrate the protective effects of antioxidant and neuroprotective drugs used in experimental studies on amikacin ototoxicity.

The major limitation of our study is the lack of histopathological examinations. Histopathological examinations could have demonstrated the protective effect of Cr against amikacin-induced ototoxicity and its non-toxic nature when used alone at the cellular level. Further studies investigating the protective effect of creatine against ototoxicity using biochemical and histopathological assessments are warranted.

## Conclusion

Our findings demonstrate that creatine treatment protects against amikacin ototoxicity when given at a sufficient dose and for an adequate time period.

## Conflicts of interest

The authors declare no conflicts of interest.
